# Tumor Necrosis Factor-*α*-Induced Colitis Increases NADPH Oxidase 1 Expression, Oxidative Stress, and Neutrophil Recruitment in the Colon: Preventive Effect of Apocynin

**DOI:** 10.1155/2014/312484

**Published:** 2014-09-04

**Authors:** Souad Mouzaoui, Bahia Djerdjouri, Nesrine Makhezer, Yolande Kroviarski, Jamel El-Benna, Pham My-Chan Dang

**Affiliations:** ^1^Institut National de la Santé et de la Recherche Médicale U1149, Equipes de Recherches Labellisées CNRS, Centre de Recherche sur l'Inflammation, 75018 Paris, France; ^2^Faculté des Sciences Biologiques, Laboratoire de Biologie Cellulaire et Moléculaire, Université des Sciences et de la Technologie Houari Boumediene, 16111 Algiers, Algeria; ^3^Université Paris Diderot, Sorbonne Paris Cité, Laboratoire d'Excellence Inflamex, Faculté de Médecine, Site Xavier Bichat, 75018 Paris, France; ^4^Assistance Publique-Hôpitaux de Paris, Centre Hospitalier Universitaire Xavier Bichat, CIB Phenogen, 75018 Paris, France

## Abstract

Reactive oxygen species- (ROS-) mediated injury has been implicated in several inflammatory disorders, including inflammatory bowel disease (IBD). NADPH oxidases (NOXs) are the major source of endogenous ROS. Here, we investigated the role of NOXs derived-ROS in a mouse model of colitis induced by the proinflammatory cytokine, tumor necrosis factor-*α* (TNF-*α*). Intraperitoneal injection of TNF*α* (10 *μ*g *·* kg^−1^) induced an acute inflammation of the colon and a marked increase in expression of NADPH oxidase 1 (NOX1), a colon specific NADPH oxidase isoform. TNF*α*-induced colitis was also characterized by high production of keratinocyte-derived chemokine (KC) and mucosal infiltration of neutrophils, NOX2-expressing cells. Concomitantly, ROS production and lipid peroxidation were significantly enhanced while catalase activity and glutathione level were reduced indicating a redox imbalance in the colon. Furthermore, the redox-sensitive MAP kinases, ERK1/2 and p38 MAPK, were activated during TNF*α*-induced colitis. Pretreatment of mice with apocynin, an NADPH oxidase inhibitor with antioxidant properties, before TNF*α* challenge, prevented all these events. These data suggest that ROS derived from NADPH oxidases (mainly NOX1 and NOX2) and MAP kinase pathways could contribute to the induction and expansion of oxidative lesions characteristics of IBD and that apocynin could potentially be beneficial in IBD treatment.

## 1. Introduction

Crohn's disease (CD) and ulcerative colitis (UC) are the two most common forms of inflammatory bowel diseases (IBD), characterized by chronic and recurrent inflammation of the gastrointestinal tract. The etiology and pathogenesis of UC and CD are multifactorial and include genetic, environmental, and immunologic factors [1–4]. Among the immunoregulatory factors, tumor necrosis factor-*α* (TNF*α*), a proinflammatory cytokine, is increased in the mucosa of IBD patients [5] and has been shown to play a central role in the pathogenesis of the disease while anti-TNF*α* therapy has been shown to be beneficial in the treatment of IBD [6, 7]. On the other hand, excessive reactive oxygen species (ROS) production has been observed in the inflamed mucosa of IBD patients [8–10]. These highly cytotoxic molecules could contribute to tissue damage in IBD [11] and could be released by activated phagocytes such as neutrophils and macrophages that are recruited in large numbers into the colonic mucosa of IBD patients [2]. Phagocytes indeed possess an enzymatic system that is dedicated to the production of ROS such as superoxide anion (O_2_^∙−^) and hydrogen peroxide (H_2_O_2_), through the single-electron reduction of molecular oxygen, with NADPH as the electron donor [12, 13]. Under nonpathological conditions, ROS produced by the phagocyte NADPH oxidase have important antimicrobial properties and are, thus, crucial for host defence against microbial infections [14]. Structure and regulation of the phagocyte NADPH oxidase are well characterized [15–18]. A functional phagocytic NADPH oxidase complex consists of the membrane-anchored flavocytochrome b558 (the catalytic core of the enzyme composed of gp91^*PHOX*^ and p22^*PHOX*^), the regulatory cytosolic proteins p47^*PHOX*^, p67^*PHOX*^, and p40^*PHOX*^, and the low molecular-weight GTP-binding proteins, Rac 1 or 2 [12]. However, the expression of the NADPH oxidase is not restricted to phagocytes. Several homologues of gp91^*PHOX*^, including NOX1, NOX3, NOX4, NOX5, DUOX1, and DUOX2, have been identified. They are encoded by separate genes, show distinctive tissue-restricted expression patterns, and are important mediators of various cellular and biological functions, such as signalling and innate immunity [19]. In particular, the colon abundantly expresses NADPH oxidase 1 (NOX1), which is the homologue most closely related to NOX2 in terms of its structure and function [20]. It interacts with p22^*PHOX*^ [21], and its activation also requires binding to regulatory partners: NOX organizer 1 (NOXO1), the p47^*PHOX*^ homologue; NOX activator 1 (NOXA1), the p67^*PHOX*^ homologue [22]; and Rac1 GTPase [23]. NOXO1 and NOXA1 transcripts are also abundantly expressed in the colon [20, 22]. Interestingly, the amount of NOX1 transcripts in the colon follows a gradient that closely parallels the bacterial burden, with intermediate level in the proximal colon and high level in the distal colon [24–26], supporting a role for NOX1 in innate immunity [27]. It is not clear whether dysregulation of NOX1 activity or expression could be linked to pathological situations in the colon such as IBD.

In the present study, we investigated the role of NADPH oxidase derived-ROS in intestinal inflammation. In particular, we examined the expression of NOX1 and the effect of apocynin (4-hydroxy-3-methoxyacetophenone), an NADPH oxidase inhibitor with antioxidant properties originally extracted from the roots of the Himalayan herb* Picrorhiza kurroa* [28], on key features associated with the mouse model of acute colitis induced by TNF*α*.

## 2. Materials and Methods

### 2.1. Reagents

Tumor necrosis factor-*α* (TNF*α*) was from PeproTech France (Neuilly-Sur-Seine, France). Apocynin (4-hydroxy-3-methoxyacetophenone), o-dianisidine hydrochloride, and thiobarbituric acid were obtained from Sigma Aldrich (Saint-Quentin Fallavier, France). Mouse KC/CXCL1 ELISA Kit was from RayBiotech, Inc. (Norcross, GA, USA). One-Step RT-PCR kit was from Qiagen S.A.S.-France (Courtaboeuf, France). Mouse anti-NOX1, -NOXA1, and -NOXO1 were a kind gift from Dr. R. Brandes (Instituts für Kardiovaskuläre Physiologie, Germany). In some experiments, rabbit polyclonal anti-NOX1 antibody from Sigma Aldrich (Saint-Quentin Fallavier, France), anti-NOXA1 antibody raised against the full-length protein from Sigma Aldrich (Saint-Quentin Fallavier, France), and anti-NOXO1 antibody directed against full-length proteins from Morphosys AbD GmbH (Düsseldorf, Germany) were also used. Anti-p22^*PHOX*^ and anti-DUOX2 (Y-15) were from Santa-Cruz Biotechnology (Heidelberg, Germany). Anti-phospho-ERK 1/2 and anti-phospho-p38MAPK antibodies were from R&D systems Europe (Abingdon, UK). Anti-phospho-JNK was from Cell Signaling Technology (Danvers, USA). Anti-ERK 1/2, anti-p38MAPK, and anti-JNK antibodies were from Santa-Cruz Biotechnology (Heidelberg, Germany).

### 2.2. Animal Care

Male NMRI (Naval Medical Research Institute) Swiss mice, weighing 20–22 g, were from the “Institut Pasteur d'Alger” (Algiers, Algeria). Mice, randomly divided into seven groups of five animals each, were kept under controlled conditions throughout the experiments. Animals were fed standard rodent chow and water* ad libitum* and kept under controlled temperature (22 ± 1°C), humidity (65–70%), and a 12 : 12 h light-dark cycle throughout the experiments. All animal work was conducted according to relevant national and international guidelines in accordance with the recommendations of the Weatherall report. All animal experiments were performed in compliance with the care and use of laboratory animals.

### 2.3. Induction of Colitis and Treatment with Apocynin

Mice were deprived of food 24 hr before colitis induction. Seven groups of five mice were used. Experimental colitis was induced in three groups by intraperitoneal injection of TNF*α* (10 *μ*g *·* kg^−1^ bw), dissolved in saline [29]. Three other groups received intraperitoneal injections of apocynin (25 mg *·* kg^−1^ bw) 30 min before TNF*α* challenge. The dose of apocynin was chosen based on previous studies showing that* in vivo* treatment with doses between 5 mg *·* kg^−1^ and 50 mg *·* kg^−1^ was efficient and safe [30, 31]. The control group received 0.9% NaCl solution. Mice pretreated or not with apocynin before TNF*α* challenge were sacrificed at 6, 18, or 36 hr after colitis induction. Control mice were sacrificed at 6, 18, or 36 hr after sterile saline administration and in some cases only at 36 hr. Colons were immediately harvested and processed for biochemical, histological, and molecular analyses, conducted in parallel.

### 2.4. Assessment of TNF*α*-Induced Macroscopic and Histological Damage

Colons were opened longitudinally and washed with ice-cold saline and blind analyses of colon macroscopic damage were performed using the following scoring [32]: (1) normal mucosa with mild hyperaemia, no ulcers; (2) linear ulcer with no significant inflammation; (3) <5 mm hemorrhagic lesions, secondary granulomatous lesion along the length of the colon; (4) two additional major sites of ulceration and hemorrhagic lesions extending >5 mm and/or perforation of the colon. Distal colonic segments were fixed in 10% formalin-PBS overnight, dehydrated in graded ethanol solutions, and embedded in paraffin. The histological samples were sectioned at 5 *μ*m, stained with hematoxylin and eosin (H&E), and analyzed by light microscopy (Carl Zeiss, Germany). Colon microscopic damage was scored as described by Neurath et al. [33]: (1) no leukocyte recruitment; (2) moderate level of leukocyte infiltration; (3) high vascular density and thickening of colon wall; (4) transmural leukocyte infiltration, loss of goblet cells, high vascular density, and thickening of colon wall.

### 2.5. RT-PCR

For RT-PCR, colon samples were preserved in RNA*later* solution (Qiagen S.A.S.-France, Courtaboeuf, France) and stored at −80°C. Total RNA was extracted from colon tissues using the RNeasy Mini Kit from Qiagen (Courtaboeuf, France) and treated with RNase-free DNase in order to remove potential genomic DNA contaminants according to manufacturer's protocol. RNA concentrations were determined by measuring the absorbance at 260 nm. RT-PCR was performed with the Qiagen One-Step RT-PCR Kit. Each sample contained an optimal amount of total RNA in 10 *μ*L as template and gene-specific primers in a final volume of 50 *μ*L. The amount of template for each gene-specific primer set was determined by generating a standard curve with various concentrations of total RNA and corresponded to a signal that was half that of the saturating signal, that is, 65 ng for NOX1, 75 ng for NOXA1, 22 ng for NOXO1, and 6 ng for p22^*PHOX*^. Mice GADPH served as internal control. The RT-PCR profile was 1 cycle of cDNA synthesis at 50°C for 30 min and 1 cycle of PCR activation step at 95°C for 15 min followed by 30 cycles of denaturation at 94°C for 30 sec, annealing at 60°C for 45 sec, extension at 72°C for 1 min, and 1 cycle of final extension at 72°C for 10 min. Aliquots of the RT-PCR products were separated on 2% agarose gel that contained 5 *μ*L of SYBR safe and resulting gels were photographed. The primer sequences were the following: NOX1, forward (TGA ACA ACA GCA CTC ACC AAT GCC), reverse (TCA TTG TCC CAC ATT GGT CTC CCA), product 431 pb; NOXA1, forward (TCT GCG CTG TGC TTC TTC TCA GAT), reverse (AGG AAA TCC ATG GGC TCC AGA TGT), product 526 pb; NOXO1, forward (CCA TGC TGT AGC CTT GGT GCA AAT), reverse (AAA CCA GGC TAC CTG CTG ATC CTT), product 598 pb; p22^*PHOX*^, forward (ATG GGG CAG AIC GAG TGG GC′C ATG), reverse (TCA CAC GAC C′FC ATC TGT CAC TGG), product 579 pb; GADPH, forward (CGT AGA CAA AAT GGT GAA GGT), reverse (GAC TCC ACG ACA TAC TCA GC), product 297 pb.

### 2.6. Protein Extraction and Western Blot Analysis

Mice colons were homogenized using rotor-stator in PBS supplemented 0.1% sodium dodecyl sulfate (SDS), 1% Triton X-100, and protease inhibitors. Protein concentration was determined by the Bradford method [34], and proteins were then boiled at 95°C in Laemmli sample buffer [35] and separated on 11% SDS-PAGE. The proteins were next electrotransferred to nitrocellulose membranes. The membranes were blocked for one hr at room temperature in TBS-T (20 mM Tris-HCl, pH 7.6, 137 mM NaCl, 0.1% Tween 20) containing 5% nonfat dry milk and then incubated overnight at 4°C with the primary antibody at the following dilutions: NOX1 (1 : 1000), p22^*PHOX*^ (1 : 1000), NOXA1 (1 : 2000), NOXO1 (1 : 2000), phospho-ERK1/2 (1 : 1000), phospho-p38MAPK (1 : 1000), and phospho-JNK (1 : 1000). After five washes of 5 min each with TBS-T, the membranes were incubated with horseradish peroxidase-conjugated goat anti-rabbit or anti-mouse antibodies for 1 hr (1 : 30,000). After extensive washing, chemiluminescence substrates were used to reveal the protein bands on Hyperfilm ECL (Amersham) or with Fusion FX7 Chemiluminescence System (Thermo Fisher Scientific). *β*-Actin, total ERK1/2, total p38MAPK, or total JNK antibodies were used as control for protein loading and transfer.

### 2.7. Measurement of KC

Colonic tissues were homogenized using rotor-stator in buffer containing 20 mM Tris-HCl pH 7.5, 250 mM saccharose, 10 mM EGTA, 2 mM EDTA, 10 *μ*g/mL leupeptin, 10 *μ*g/mL pepstatin, 10 *μ*g/mL aprotinin, and 1 mM PMSF. The homogenates were centrifuged at 100,000 g for 1 hr at 4°C. KC level was measured in the postcentrifugation supernatant using a commercially available Mouse KC ELISA kit (Ray Biotech, Inc., USA). Recombinant mouse KC was used as standard. Briefly, plates were incubated overnight at 4°C with 100 *μ*L of standard or sample into appropriate wells and washed; then, 100 *μ*L of biotinylated antibody was added to each well and incubated for 1 hr at room temperature. After washing, plates were incubated with a Streptavidin solution. After 45 min, wells were washed again and the color developed with TMB One-Step Substrate (3,3′,5,5′-tetramethylbenzidine) for 30 min in the dark. The reaction was stopped by addition of 2 M sulfuric acid. Optical densities were determined at 450 nm, and the concentration of KC was calculated as pg of KC *·* *μ*g^−1^ of protein.

### 2.8. Myeloperoxidase Assay

Myeloperoxidase (MPO) activity was used as an index of neutrophil accumulation in the inflamed colon and was determined as previously described [36]. Briefly, distal colonic segments were homogenized in ice-cold 50 mM phosphate buffer (pH 7.2), containing 0.5% Triton X-100. After three freeze-thawed cycles, the homogenates were centrifuged at 10,000 g for 10 min at 4°C. The resulting supernatant (0.1 mL) was added to 2.9 mL of 50 mM phosphate buffer pH 6.0, containing 0.167 mg/mL o-dianisidine hydrochloride. The reaction was started with addition of 0.0005% hydrogen peroxide and the change in absorbance at 460 nm was recorded for 5 min at 30°C. MPO activity was expressed as *μ*M H_2_O_2_ *·* min^−1^ *·* mg^−1^ protein.

### 2.9. Malondialdehyde Content

Malondialdehyde (MDA) content was used as an indicator of lipid peroxidation [37]. Colon tissues were homogenized in 1.15% KCl and centrifuged at 10,000 g for 10 min at 4°C. The resulting supernatants (0.1 mL) were boiled for 1 hr at 95°C in a 4 mL final volume of reaction mixture containing 8.1% SDS (0.2 mL), 20% acetic acid (1.5 mL), 0.8% thiobarbituric acid (1.5 mL), and distilled water (0.7 mL). The reaction was stopped in an ice bath and the supernatants were recovered by centrifugation at 3000 g for 10 min. The absorbance of the supernatants was measured by spectrophotometry at 532 nm. Results were expressed as nmol MDA *·* mg^−1^ protein (molar extinction coefficient: 156 × 10^2^ M^−1 ^cm^−1^).

### 2.10. Nitroblue Tetrazolium (NBT) Reduction Assay

Superoxide anion production was evaluated by NBT reduction measured in fresh tissues [38]. Briefly, 100 mg of distal colonic tissues from control or mice with TNF*α*-induced colitis, pretreated or not with apocynin, was incubated for 30 min at 37°C in D-glucose phosphate-buffered saline (D-PBS) in the presence of 2 mg *·* mL^−1^ NBT. After tissue homogenization and centrifugation, reduced NBT was solubilized in 50% (v/v) acetic acid. The absorbance of reduced NBT was determined at 560 nm.

### 2.11. Catalase Assay

Catalase activity in the colon was determined by the method of Aebi [39]. The principle of the assay is based on the determination of the rate constant of hydrogen peroxide decomposition by the catalase enzyme. Decomposition of H_2_O_2_ was followed directly by monitoring the decrease of absorbance at 240 nm. Data were expressed as unit per mg^−1^ protein.

### 2.12. Reduced Glutathione Content

Glutathione (GSH) level in the colon was measured by the method of Ellman [40]. The GSH levels were estimated by the reduction of DTNB (dithiobis-2-nitrobenzoic acid). Tissue homogenates were deproteinized and centrifuged at 600 g for 20 min. After addition of DTNB and phosphate buffer (pH 8.0) into the cleared sample supernatants, the yellow color developed was read at 412 nm. Results were expressed as nmol of GSH mg^−1^ protein (molar extinction coefficient: 1.36 × 10^4^ M^−1 ^cm^−1^).

### 2.13. Statistical Analysis

All results are expressed as means ± SEM. Significant differences were identified with Student's *t*-test and by one-way ANOVA followed by Scheffe's post hoc test when multiple variables were analyzed (significance threshold *P* < 0.05). Histological damage scores were analyzed by Mann-Whitney's *U* test.

## 3. Results

### 3.1. Apocynin Prevents the Colon Damage Induced by TNF*α*

**Figure 1:**
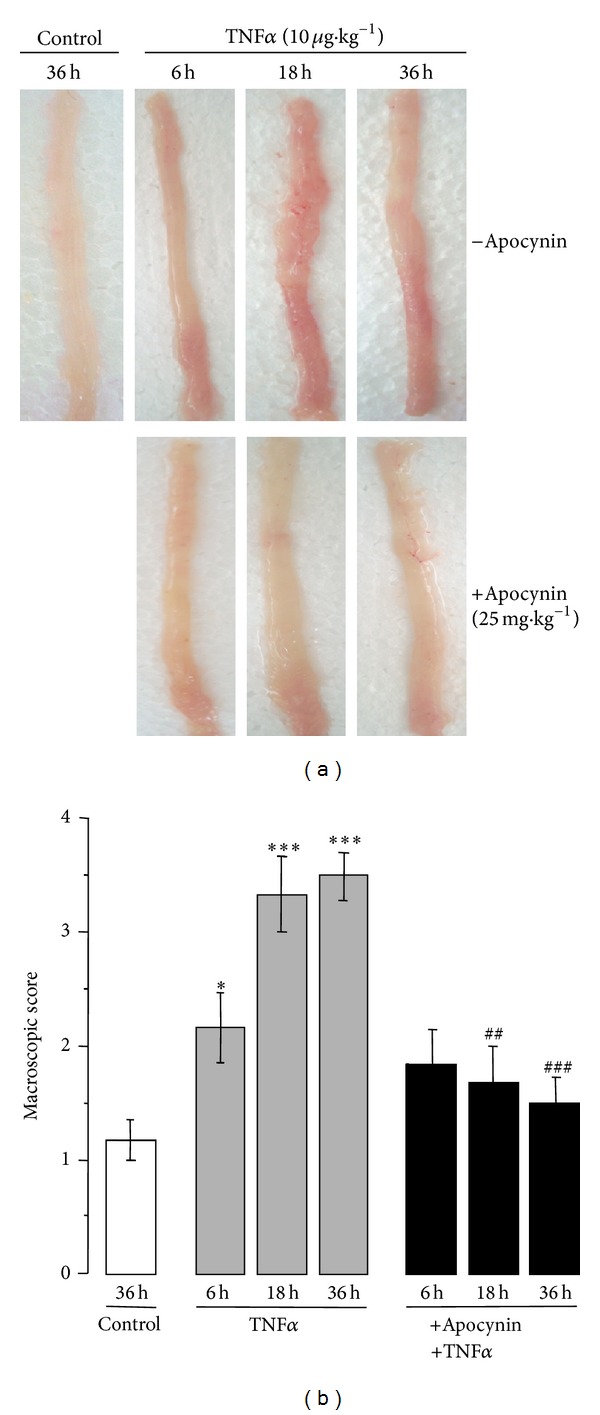
Apocynin improves TNF*α*-induced macroscopic damage of the colon. (a) Macroscopic examination of the colon of mice treated with TNF*α* (10 *μ*g *·* kg^−1^, ip) in the presence or absence of apocynin (25 mg *·* kg^−1^, ip). Apocynin was given 30 min before TNF*α* administration. Colons were resected at 6, 18, and 36 hr after TNF*α* treatment. Control mice were given saline and sacrificed at 36 hr; (b) macroscopic score damage in the colon of mice treated with TNF*α* (10 *μ*g *·* kg^−1^, ip) in the presence or absence of apocynin (25 mg *·* kg^−1^, ip). Results from mice treated with 10 *μ*g kg^−1^ TNF*α* were matched to control mice with Mann-Whitney's *U* test. Values are means ± SEM from *n* = 5 mice in each group. *P < 0.05 and ***P < 0.001 versus control group; ^##^ *P* < 0.01 and ^###^ *P* < 0.001 versus TNF*α* treated group.

**Figure 2:**
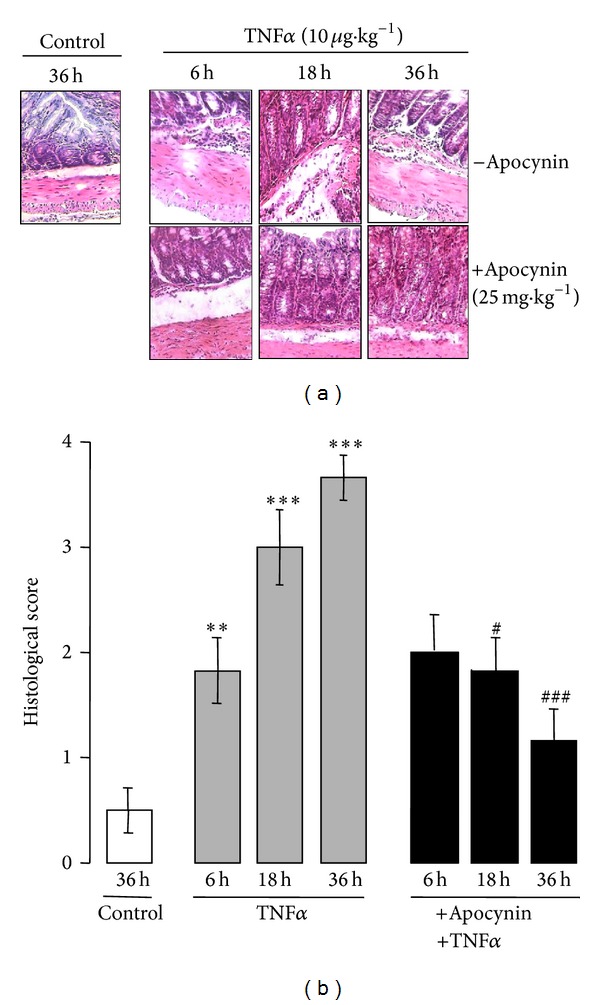
Apocynin improves TNF*α*-induced histological changes of the colon. (a) Histological examination of the colon sections of mice treated with TNF*α* (10 *μ*g *·* kg^−1^, ip) in the presence or absence of apocynin (25 mg *·* kg^−1^, ip). Mice were treated as described in Figure 1(a), and colon sections were stained with hematoxylin and eosin (H&E) and analyzed by light microscopy (Gx400); (b) histological score damage in the colon of mice treated with TNF*α* (10 *μ*g *·* kg^−1^, ip) in the presence or absence of apocynin (25 mg *·* kg^−1^, ip). Results from mice treated with 10 *μ*g kg^−1^ TNF*α* were matched to control mice with Mann-Whitney's *U* test. Values are means ± SEM from *n* = 5 mice in each group. **P < 0.01 and ***P < 0.001 versus control group; ^#^ *P* < 0.05 and ^###^ *P* < 0.001 versus TNF*α*-treated group.

Intraperitoneal injection of TNF*α* (10 *μ*g *·* kg^−1^) in Swiss mice induced several clinical symptoms and morphological alterations, including an increase in colonic weight and stool softening as well as a significant decrease in colonic length (Table 1). An increase in splenic mass was also observed (Table 1). No rectal bleeding was observed (not shown). In addition, as shown in our previous study [29], macroscopic examination of the colon revealed that intraperitoneal administration of TNF*α* caused severe damage of the colonic mucosa as shown by the presence of numerous mucosal erosions and hemorrhagic areas (Figure 1(a)). In contrast, the colons of control mice treated with saline were normal (Figure 1(a)). Colonic lesions usually occurred 6 hr following TNF*α* challenge, with moderate damage and a macroscopic score of 2.16 ± 0.33. The damage was greater with severe hemorrhagic lesions after 18 and 36 hr, with a macroscopic damage score of 3.33 ± 0.36 and 3.5 ± 0.24, respectively (Figures 1(a) and 1(b)). In comparison, control mice treated with saline for 36 hr had a macroscopic score of 1.17 ± 0.18 (Figure 1(b)). Histological examination of colon sections from TNF*α*-treated mice stained with hematoxylin-eosin (H&E) showed loss of crypts in the mucosa membrane and abundant infiltration of inflammatory cells within the under mucous layer (Figure 2(a)) as compared to the colon sections from control mice. The histological damage scores following TNF*α* treatment were 1.83 ± 0.33, 3 ± 0.4, and 3.66 ± 0.23 at 6, 18, and 36 hr, respectively (Figure 2(b)), whereas those of control mice were 0.5 ± 0.24. Interestingly, pretreatment of mice 30 min before TNF*α* challenge with apocynin, an NADPH oxidase inhibitor that also possesses ROS scavenging properties [28], significantly attenuated the extent and severity of the colon injury caused by TNF*α*, as reflected by a decrease in the morphological alterations (Table 1) and the reduction of the macroscopic scores (Figures 1(a) and 1(b)). In addition, apocynin clearly prevented the morphological signs of cell damage and the infiltration of inflammatory cells induced by TNF*α* as reflected by the significant reduction of histological scores (Figures 2(a) and 2(b)). Thus, the prevention of TNF*α*-induced injury by apocynin, an inhibitor of the NADPH oxidases with antioxidant properties [28], suggests the involvement of ROS.

### 3.2. Expression of NOX1, NOXA1, NOXO1, and p22^*PHOX*^ ****Was Increased in the Colon of TNF*α*-Treated Mice but Was Attenuated by Apocynin

**Figure 3:**
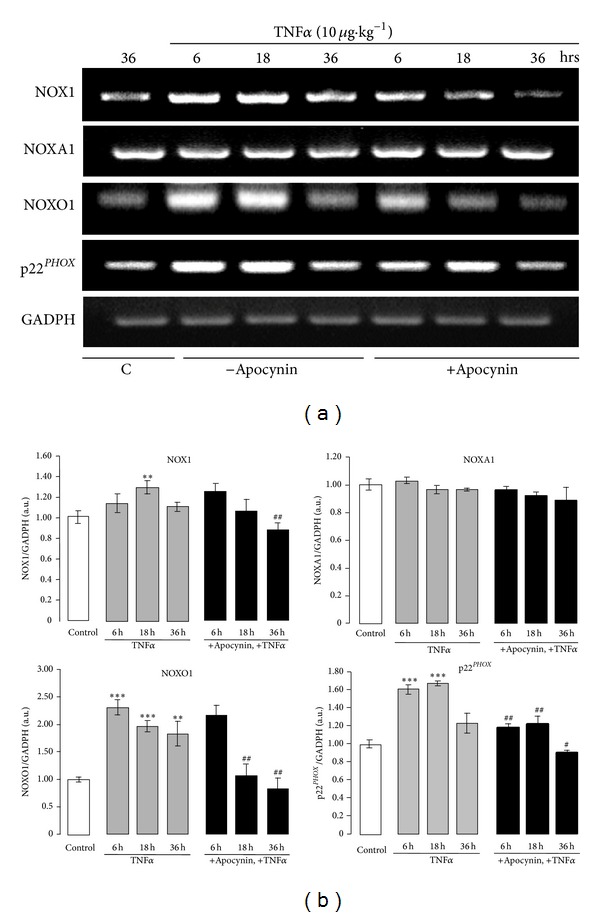
mRNA expression of NOX1, NOXA1, NOXO1, and p22^*PHOX*^ in the colon of TNF*α*-treated mice: effect of apocynin. (a) Mice were treated with TNF*α* (10 *μ*g *·* kg^−1^, ip) in the presence or absence of apocynin (25 mg *·* kg^−1^, ip) as described in Figure 1(a) (C in the figure is for control untreated mice). Total RNA from colons was extracted and RT-PCR was performed as described in Section 2 using NOX1, NOXA1, NOXO1, or p22^*PHOX*^ gene-specific primers. Mice GADPH served as an internal control; (b) densitometric analysis of the ratio of NOX1, NOXA1, NOXO1, or p22^*PHOX*^ mRNA expression to the total amount of GADPH mRNA. Data are expressed as means ± S.E.M from *n* = 5 mice in each group. *P < 0.05, **P < 0.01, and ***P < 0.001 versus control; ^#^ *P* < 0.05, ^##^ *P* < 0.01, and ^###^ *P* < 0.001 versus TNF*α*.

**Figure 4:**
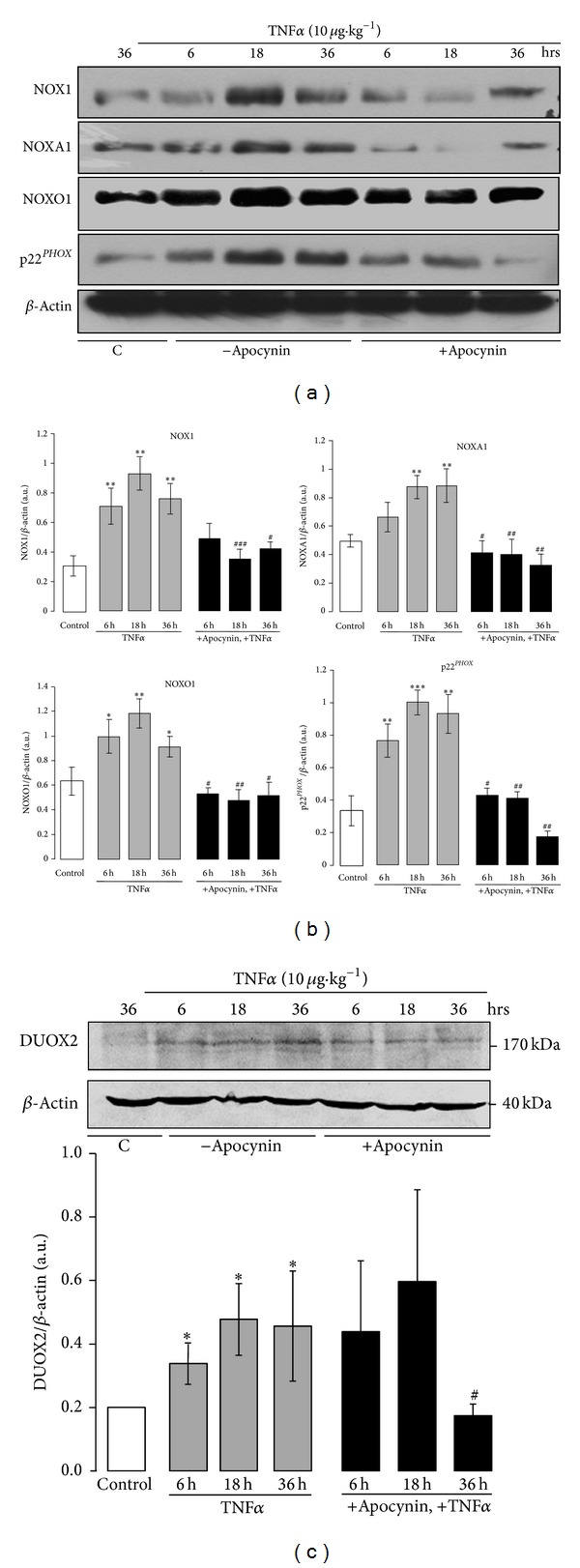
Protein expression of NOX1, NOXA1, NOXO1, and p22^*PHOX*^ in the colon of TNF*α*-treated mice: effect of apocynin. (a) Mice were treated with TNF*α* (10 *μ*g *·* kg^−1^, ip) in the presence or absence of apocynin (25 mg *·* kg^−1^, ip) as described in Figure 1(a) (C in the figure is for control untreated mice). Proteins were extracted and 50 *μ*g was subjected to SDS-PAGE and Western blot as described in Section 2 using NOX1, NOXA1, NOXO1, or p22^*PHOX*^ specific antibodies. Mice *β*-actin served as a control for protein loading and transfer; (b) densitometric analysis of the ratio of NOX1, NOXA1, NOXO1, or p22^*PHOX*^ protein expression to the total amount of *β*-actin. Data are expressed as means ± S.E.M from *n* = 5 mice in each group. *P < 0.05, **P < 0.01, and ***P < 0.001 versus control; ^#^ *P* < 0.05, ^##^ *P* < 0.01, and ^###^ *P* < 0.001 versus TNF*α*. (c)* Upper: *Western blot analysis of DUOX2 expression using specific antibodies. Mice *β*-actin served as a control for protein loading and transfer.* Lower: *densitometric analysis of the ratio of DUOX2 protein expression to the total amount of *β*-actin. Data are expressed as means ± S.E.M from *n* = 5 mice in each group. *P < 0.05 versus control; ^#^ *P* < 0.05 versus TNF*α*.

We next examined the effect of TNF*α* on the expression of the ROS-generating enzymes in the colon. We focused on the NOX1 enzyme complex, as it is the most abundantly expressed in the colon [20, 22].  Figure 3 shows the basal expression of NOX1, NOXA1, NOXO1, and p22^*PHOX*^ mRNA in the colon. Interestingly, RT-PCR experiments demonstrated that TNF*α*-induced colon damage was associated with an increase of the mRNA levels of NOX1, NOXO1, and p22^*PHOX*^ as compared to untreated control mice (Figures 3(a) and 3(b)). In contrast, the mRNA level of the internal control gene, GADPH, did not vary, indicating specificity of the increase. Semi-quantitative analysis by densitometry showed a maximal increase of NOX1 mRNA expression 18 hr after TNF*α* challenge and a maximal increase of NOXO1 and p22^*PHOX*^ mRNA expression 6 hr after TNF*α* challenge (Figure 3(b)). In contrast, no significant increase of NOXA1 mRNA expression was detected in the colons at any time (Figures 3(a) and 3(b)). Expression of NOX1, NOXA1, NOXO1, and p22^*PHOX*^ was then analyzed at the protein level. Concomitant to the increase in mRNA, protein expression of NOX1, p22^*PHOX*^, and NOXO1 was increased at 6, 18, and 36 hr following the treatment of mice with TNF*α* as compared to untreated control mice (Figures 4(a) and 4(b)). Although no increase in NOXA1 mRNA expression was observed in the colon of TNF*α*-treated mice, NOXA1 protein expression was clearly increased at 6, 18, and 36 hr after TNF*α* challenge (Figures 4(a) and 4(b)).

The administration of apocynin significantly inhibited TNF*α*-induced increase of NOX1, NOXO1, and p22^*PHOX*^ expression in the colon at the mRNA and protein levels and TNF*α*-induced increase of NOXA1 at the protein level (Figures 3(a) and 3(b); Figures 4(a) and 4(b)). As DUOX2 homologue of NADPH oxidase is known to be expressed in the colon [41], although at a lower level than NOX1, we next investigated DUOX2 expression during TNF*α*-induced colitis. A weak basal expression of DUOX2 was observed in the colon of untreated mice (Figure 4(c)). Treatment of mice with TNF*α* increased DUOX2 expression, although to a lesser extent than NOX1 expression (Figure 4(c)). Administration of apocynin prevented DUOX2 increased expression only at 36 hr.

### 3.3. Apocynin Decreased Neutrophil Infiltration and KC Level in the Colon of TNF*α*-Treated Mice

**Figure 5:**
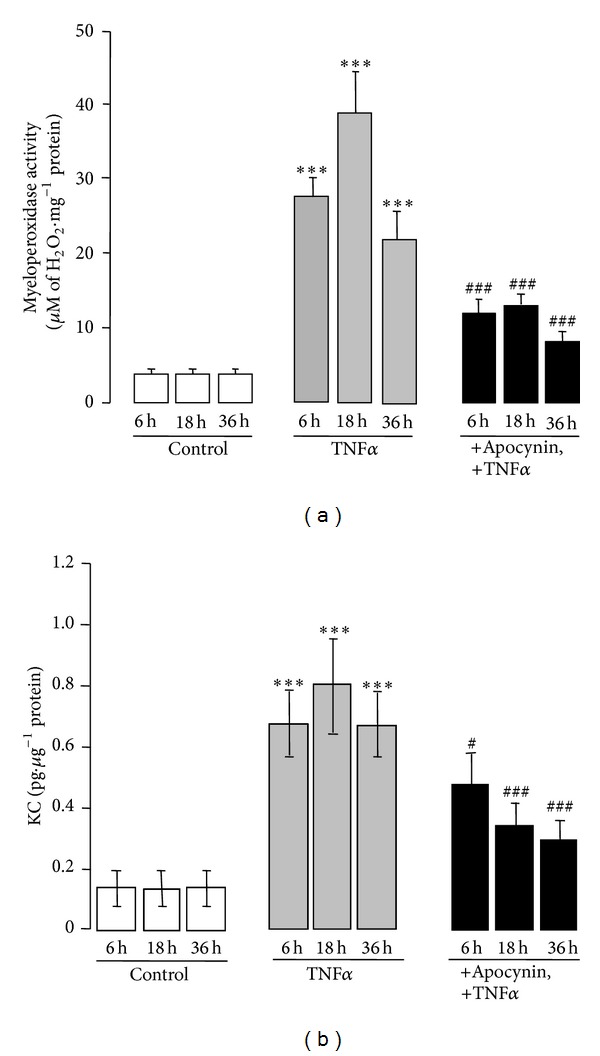
Apocynin decreases neutrophils infiltration and KC level in the colon of TNF*α*-treated mice. (a) Neutrophils infiltration was determined by measuring MPO activity, as described in Section 2, in the colon of mice treated with TNF*α* (10 *μ*g *·* kg^−1^, ip) in the presence or absence of apocynin (25 mg *·* kg^−1^, ip). Data are expressed as means ± S.E.M from *n* = 5 mice in each group. *P < 0.05, **P < 0.01, and ***P < 0.001 versus control; ^#^ *P* < 0.05, ^##^ *P* < 0.01, and ^###^ *P* < 0.001 versus TNF*α*; (b) KC level was measured by ELISA in the colon of mice treated with TNF*α* (10 *μ*g *·* kg^−1^, ip) in the presence or absence of apocynin (25 mg *·* kg^−1^, ip) as described in Section 2. Data are expressed as means ± S.E.M from *n* = 5 mice in each group. *P < 0.05, **P < 0.01, and ***P < 0.001 versus control; ^#^ *P* < 0.05, ^##^ *P* < 0.01, and ^###^ *P* < 0.001 versus TNF*α*.

TNF*α*-induced colitis was characterized by a substantial neutrophil infiltration, as shown by the presence of MPO activity in the inflamed colon. MPO activity increased up to 7- to 10-fold after 6, 18, and 36 hr of TNF*α* administration (***P < 0.001) as compared to control untreated mice (Figure 5(a)). Interestingly, this neutrophil influx was associated with the detection of high levels of keratinocyte-derived chemokine (KC), a chemoattractant cytokine for neutrophils (Figure 5(b)). The KC concentration increased up to 5-fold between 6 and 36 hr after TNF*α* challenge, peaking after 18 hr at 6-fold (***P < 0.001), as compared to control mice. Interestingly, administration of apocynin to TNF*α*-treated mice reduced by more than 50% the MPO activity induced 6 and 18 hr after TNF*α* challenge (^###^ *P* < 0.001), thereby showing a markedly attenuated neutrophil infiltration into the colon. This decrease was even greater 36 hr after colitis induction (Figure 5(a)). In addition, the decrease in neutrophil infiltration closely correlated with the reduced levels of KC in the TNF*α*-treated mice that received apocynin. KC levels were decreased by 30% (^#^ *P* < 0.05) 6 hr after colitis induction by TNF*α* and by more than half 18 and 36 h after TNF*α* treatment (^###^ *P* < 0.001). These findings confirm the enhanced infiltration of inflammatory cells in the colon of TNF*α*-treated mice observed by histology and suggest that the neutrophil influx could be induced by KC.

### 3.4. TNF*α*-Induced Colitis Resulted in a Redox Imbalance in the Colon, an Effect That Was Prevented by Apocynin

**Figure 6:**
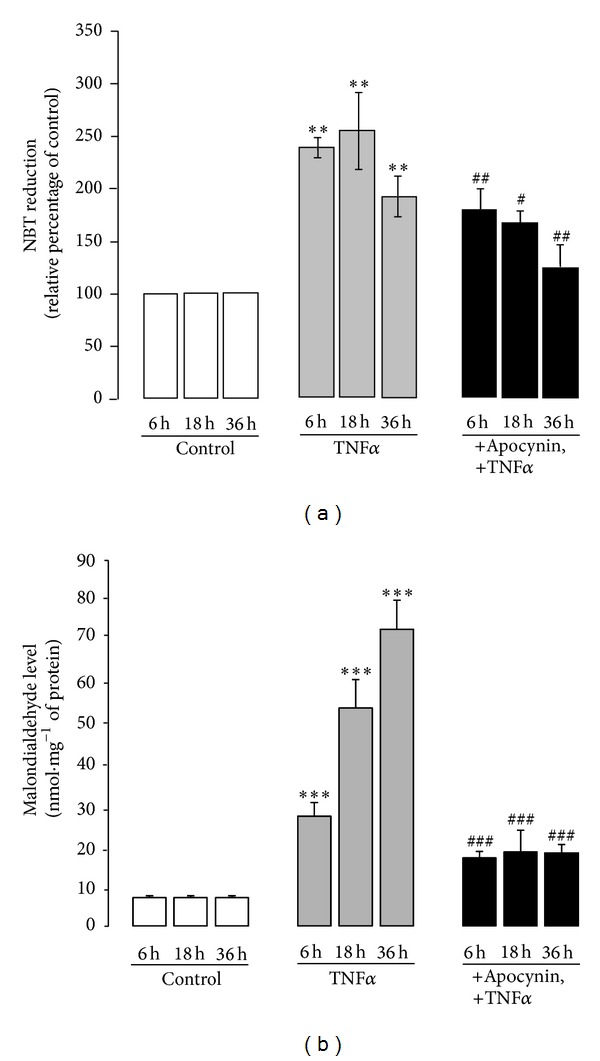
TNF*α*-induced colitis results in an increase of oxidative stress markers in the colon, an effect that is prevented by apocynin. (a) Superoxide anion release was evaluated by NBT reduction in fresh colon tissues of mice treated with TNF*α* (10 *μ*g *·* kg^−1^, ip) in the presence or absence of apocynin (25 mg *·* kg^−1^, ip) as described in Section 2. Data are expressed as percentage of control absorbance at 560 nm (control mice treated with saline). Means ± S.E.M from *n* = 5 mice in each group. ***P < 0.001 versus control group; and ^#^ *P* < 0.05; ^###^ *P* < 0.001 versus TNF*α*-treated group; (b) lipid peroxidation was assessed by malondialdehyde (MDA) levels in fresh colon tissues of mice treated with TNF*α* (10 *μ*g *·* kg^−1^, ip) in the presence or absence of apocynin (25 mg *·* kg^−1^, ip) as described in Section 2. Data are expressed as means ± S.E.M from *n* = 5 mice in each group. *P < 0.05, **P < 0.01, and ***P < 0.001 versus control; ^#^ *P* < 0.05, ^##^ *P* < 0.01, and ^###^ *P* < 0.001 versus TNF*α*.

As TNF*α*-induced colitis was accompanied by a substantial increase in expression of the NOX1 subunits and an infiltration of neutrophils which could release large amount of ROS* via *NOX2, we next examined oxidative stress markers in the colon. ROS release, evaluated by NBT reduction in fresh colon tissues, was clearly increased in TNF*α*-treated mice as compared to control mice (Figure 6(a)). Moreover, malondialdehyde (MDA), an indicator of lipid peroxidation, was also strongly increased in TNF*α*-treated mice by 4-, 8-, and 10-fold after 6, 18, and 36 hr of TNF*α* treatment, respectively, as compared to control mice (Figure 6(b)). In contrast, these two markers were significantly decreased in mice treated with apocynin before the induction of inflammation by TNF*α* (Figures 6(a) and 6(b)), suggesting that apocynin, by inhibiting ROS production, protected the colon against lipid peroxidation.

**Figure 7:**
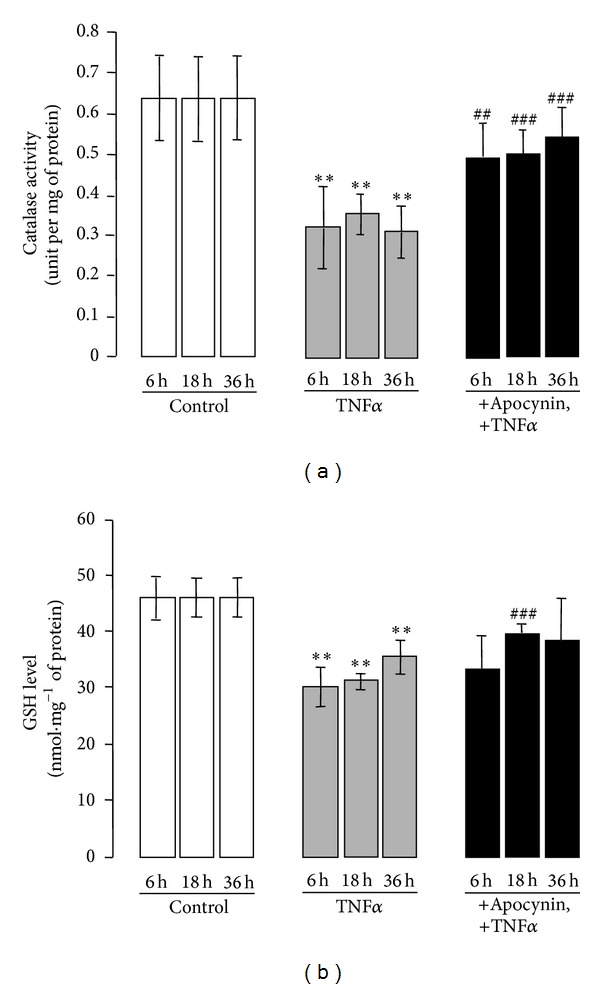
TNF*α*-induced colitis results in a decrease of endogenous antioxidants in the colon, an effect that is prevented by apocynin. (a) Catalase activity was assayed in fresh colon tissues of mice treated with TNF*α* (10 *μ*g *·* kg^−1^, ip) in the presence or absence of apocynin (25 mg*·*kg^−1^, ip) as described in Section 2. Data are expressed as means ± S.E.M from *n* = 5 mice in each group. *P < 0.05, **P < 0.01, and ***P < 0.001 versus control; ^#^ *P* < 0.05, ^##^ *P* < 0.01, and ^###^ *P* < 0.001 versus TNF*α*; (b) GSH contents were assayed in fresh colon tissues of mice treated with TNF*α* (10 *μ*g *·* kg^−1^, ip) in the presence or absence of apocynin (25 mg *·* kg^−1^, ip) as described in Section 2. Data are expressed as means ± S.E.M from *n* = 5 mice in each group. *P < 0.05, **P < 0.01, and ***P < 0.001 versus control; ^#^ *P* < 0.05, ^##^ *P* < 0.01, and ^###^ *P* < 0.001 versus TNF*α*.

Concomitantly, the antioxidative markers, catalase activity and glutathione (GSH) level, measured in colon tissues were significantly lower in the TNF*α*-treated mice compared to control mice (Figures 7(a) and 7(b)). Interestingly, administration of apocynin to mice before TNF*α* injection restored catalase activity and GSH content to control levels (Figures 7(a) and 7(b)). These data indicate a redox imbalance in the colon of TNF*α*-treated mice that is prevented by apocynin administration.

### 3.5. Redox-Sensitive MAP Kinases Are Activated in the Colon of TNF*α*-Treated Mice and Modulated by Apocynin

**Figure 8:**
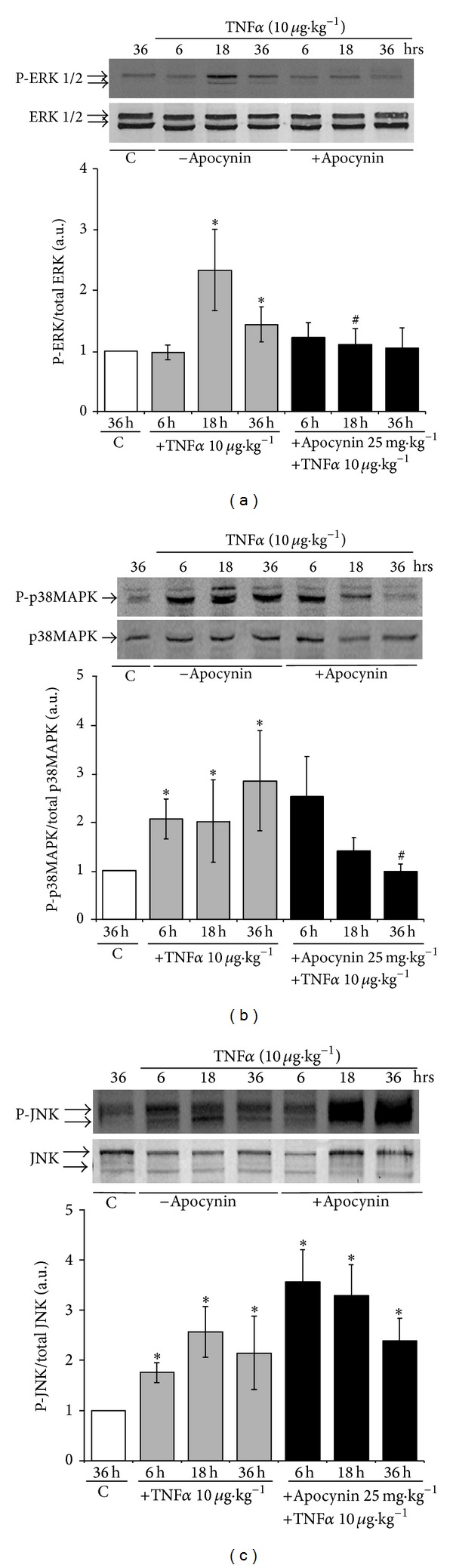
Redox-sensitive MAP kinases are activated in the colon of TNF*α*-treated mice: modulation by apocynin. (a)* Upper:* ERK1/2 activation was assessed by Western blot using phospho-ERK1/2 specific antibodies in colon of mice treated with TNF*α* (10 *μ*g *·* kg^−1^, ip) in the presence or absence of apocynin (25 mg *·* kg^−1^, ip) (C in the figure is for control untreated mice). Total ERK1/2 protein, assessed with an ERK1/2 antibody, was used as loading control.* Lower:* densitometric analysis of the ratio of phospho-ERK1/2 to total ERK1/2 protein. Data are expressed as means ± S.E.M from *n* = 5 mice in each group. *P < 0.05, **P < 0.01, and ***P < 0.001 versus control; ^#^ *P* < 0.05, ^##^ *P* < 0.01, and ^###^ *P* < 0.001 versus TNF*α*. (b)* Upper:* p38MAPK activation was assessed by Western blot using phospho-p38MAPK specific antibodies in colon of mice treated with TNF*α* (10 *μ*g *·* kg^−1^, ip) in the presence or absence of apocynin (25 mg *·* kg^−1^, ip) (C in the figure is for control untreated mice). Total p38MAPK protein, assessed with a p38MAPK antibody, was used as loading control.* Lower:* densitometric analysis of the ratio of phospho-p38MAPK to total p38MAPK protein. Data are expressed as means ± S.E.M from *n* = 5 mice in each group. *P < 0.05, **P < 0.01, and ***P < 0.001 versus control; ^#^ *P* < 0.05, ^##^ *P* < 0.01, and ^###^ *P* < 0.001 versus TNF*α*. (c)* Upper:* JNK activation was assessed by Western blot using phospho-JNK specific antibodies in colon of mice treated with TNF*α* (10 *μ*g *·* kg^−1^, ip) in the presence or absence of apocynin (25 mg *·* kg^−1^, ip) (C in the figure is for control untreated mice). Total JNK protein, assessed with a JNK antibody, was used as loading control.* Lower:* densitometry analysis of the ratio of phospho-JNK to total JNK. Data are expressed as means ± S.E.M from *n* = 5 mice in each group. *P < 0.05, **P < 0.01, and ***P < 0.001 versus control; ^#^ *P* < 0.05, ^##^ *P* < 0.01, and ^###^ *P* < 0.001 versus TNF*α*.

In order to determine if the redox imbalance resulted into the activation of redox-sensitive kinases, such as the MAP kinases, we next investigated the activation status of ERK1/2, p38MAPK, and JNK in TNF*α*-treated mice as compared to control mice through the analysis of their phosphorylation state. Treatment of mice with TNF*α* induced the phosphorylation of ERK1/2 and p38MAPK as well as JNK (Figure 8). Phosphorylation of p38MAPK and JNK occurred 6 hr following TNF*α* treatment, an effect which lasted up to 36 hr (Figures 8(b) and 8(c)), whereas activation of ERK1/2 occurred only after 18 hr (Figure 8(a)). Apocynin treatment significantly reduced TNF*α*-induced ERK1/2 and p38MAPK phosphorylation in the colon (Figures 8(a) and 8(b)). In contrast, apocynin did not prevent JNK phosphorylation, but rather increased it (Figure 8(c)). Analysis of total ERK1/2, p38MAPK, and JNK showed that the same amount of proteins was loaded into each well (Figures 8(a), 8(b), and 8(c)). These results show that the oxidative stress and the redox imbalance that occurred during TNF*α*-induced colitis are associated with an increase in the activities of the redox-sensitive MAP kinases, ERK1/2, p38MAPK, and JNK in the colon, and that only the activation of p38MAPK and ERK1/2 was prevented by the NADPH oxidase inhibitor and ROS scavenger, apocynin.

## 4. Discussion

This study demonstrates for the first time that TNF*α*-induced colitis triggers a marked increase in the expression of key components of the colon-specific NADPH oxidase isoform, NOX1, NOXA1, NOXO1, and p22^*PHOX*^. DUOX2 was also increased, however, to a lower extent than NOX1. This event was associated with high production of KC and infiltration of neutrophils, NOX2-expressing cells, in the intestinal mucosa. Concomitantly, lipid peroxidation and superoxide production were significantly enhanced while GSH and catalase activity were reduced, indicating a redox imbalance in the colon. In addition, the redox-sensitive MAP kinases, ERK1/2 and p38MAPK, were activated during TNF*α*-induced colitis. Interestingly, apocynin, a known NADPH oxidase inhibitor with antioxidant properties [28], which can inhibit both NOX1 and NOX2, prevented all these events. These data suggest that ROS derived from NADPH oxidases (mainly NOX1 and NOX2) and MAP kinase pathways could contribute to the induction and expansion of oxidative lesions characteristics of IBD and that apocynin could potentially be beneficial in IBD treatment.

The involvement of oxidative stress in the onset and/or progression of IBD is well recognized [10, 41]. ROS, such as superoxide anion (O_2_^∙−^), hydrogen peroxide (H_2_O_2_), and hypochlorite (HOCl), have been demonstrated to induce injury to epithelial cells from the inflamed mucosa in IBD patients [10], and* in vitro* studies have also demonstrated the damaging effect of enterocyte exposure to O_2_^∙−^ [42]. Excessive production and accumulation of ROS, which are highly unstable and reactive molecules, may induce tissue injury through the oxidative damage of cellular macromolecules resulting in lipid peroxidation and DNA and protein oxidation [43]. The source of ROS in IBD has been mainly attributed to activated macrophages and neutrophils, which are massively recruited into the inflamed gut [44]. These activated inflammatory cells can indeed generate large amounts of ROS through NOX2, a professional enzymatic complex that releases large quantities of O_2_^∙−^, the precursor of other ROS (OH^∙^, H_2_O_2_, and HOCl). Interestingly, our study suggests that NOX1, which is highly expressed in colon epithelial cells [25, 26], might also be another source of ROS during IBD. Expression of all of the subunits forming the NOX1 system, namely, NOX1, p22^*PHOX*^, NOXA1, and NOXO1, was indeed increased in the mouse model of TNF*α*-induced colitis. DUOX2 expression was also increased; however, this increase was moderate as compared to that of NOX1, suggesting a predominant role of NOX1 in TNF*α*-induced colitis. We cannot exclude the possibility that there might be other sources of ROS generation during inflammation of the colon. In fact, a recent study has shown that mitochondria could be a source of ROS during Crohn's disease as the mitochondrial membrane potential is inhibited during active Crohn's disease [45]. However, as apocynin, a selective inhibitor of NADPH oxidases (and not of mitochondria), has a protective effect in our model of intestinal inflammation, we believe that NADPH oxidase homologs play a predominant role in our model.

As the amount of ROS produced by NOX1 is much lower than that produced by NOX2 of neutrophils and macrophages, accounting for only 0.5 to 5 percent [19, 22, 46], it is unlikely that NOX1-derived-ROS are directly involved in tissue damage in IBD. However, it has been demonstrated that low level of ROS may act as second messengers, modulating intracellular signaling pathways through the activation of redox-sensitive kinases such as MAP kinases [47]. This pathway is able to regulate proinflammatory gene expression, including cytokines, chemokines, and adhesion molecules. Therefore, it is possible that NOX1-derived-ROS might participate in the onset of IBD by regulating activation of MAP kinases, which in turn controls proinflammatory cytokine production. It is interesting to note that a direct activation of MAPK kinases by NOX1 has been demonstrated [48, 49] and that a sustained activation of MAP kinases was observed in the inflamed mucosa of IBD patients [50]. In addition, our data show that ROS are directly involved in TNF*α*-induced activation of MAPK in the colon, as apocynin prevented the phosphorylation of p38MAPK and ERK1/2. However, it remains to be determined whether ROS produced by NOX1 or NOX2 are responsible for this effect as apocynin can inhibit both homologues. Interestingly, the upregulation of NOX1 subunits expression was associated with elevated mucosal infiltration of neutrophils as demonstrated by the increase in MPO activity and the high levels of the neutrophil chemoattractant, KC, the mouse counterpart of human IL-8. Again, both events were inhibited by apocynin indicating a ROS-dependent effect. On the other hand, we cannot exclude the possibility that the increase in NOX1 subunits expression during TNF*α*-induced colitis might actually reflect a protective response to tissue damage as a recent study demonstrated that NOX1-dependent redox signaling pathway could promote intestinal mucosal wound repair [51].

Along with the increased ROS production, we show that the levels of endogenous antioxidants in colonic tissue, that is, glutathione and catalase, were decreased in our experimental model of TNF*α*-induced colitis. It is interesting to note that antioxidant defense mechanisms, such as SOD activity and total glutathione level, were found to be decreased in inflamed mucosa of patients with UC and CD as compared to areas with noninflamed mucosa [52, 53]. Therefore, increased ROS production in combination with reduced total antioxidant capacity could cause severe oxidative stress in the chronically inflamed colon mucosa of IBD patients. Thus, it is reasonable to think that an antioxidant therapy would be beneficial for IBD treatment. Our study demonstrates that apocynin treatment resulted in a marked improvement of the damage score and lower MPO activity in TNF*α*-induced colitis. Interestingly, we further show that apocynin also concomitantly decreases the expression of NOX1 subunits, ROS production, MAP kinase activation, and KC production. The fact that apocynin inhibits mRNA and protein expression of NOX1 subunits suggests that ROS could regulate the expression of these subunits in a positive control feedback loop. Taking into account the positive effects of apocynin on all these parameters and its very low toxicity [28], this component may be useful for the treatment of IBD. However, TNF*α* which is a dominant player in the pathogenesis of IBD [54] induces an acute process of inflammation, which probably only mimics the active phase of colitis. Therefore, effectiveness of apocynin might be limited to the acute phase of the disease. We plan in future experiments to investigate if apocynin has a protective effect on a chronic animal model of the disease such as chronic model based on repeated administration of 2,4,6-trinitrobenzene sulfonic ACID (TNBS).

The observation that the expression of NOX1, NOXA1, NOXO1, and p22^*PHOX*^ is increased during TNF*α*-induced colitis is in agreement with previous* in vitro* studies showing that treatment of T84 colon epithelial cells with TNF*α* increased NOX1 and NOXO1 expression at the mRNA and protein levels [46, 55]. Our data further demonstrate that TNF*α* acts as a potent NOX1 inducer* in vivo* in the colon, suggesting that NOX1 may play a central role in the development of IBD.

Overall, our data show that key features associated with TNF*α*-induced colitis, including production of KC, high infiltration of neutrophils, redox imbalance, and activation of redox-sensitive MAP kinases, could be mediated through ROS produced by NOX1 and NOX2 from epithelial cells and neutrophils, respectively, and that these processes can be inhibited by apocynin, an NADPH oxidase inhibitor with antioxidant properties.

## Figures and Tables

**Table 1:** TNF*α*-induced colitis results in morphological alterations of the colon and spleen, an effect that is prevented by apocynin.

Group	Colon weight (mg)	Colon length (cm)	Spleen weight (mg)
Control	204 ± 19	8.1 ± 0.3	105 ± 10
TNF*α* (6 hr)	311 ± 16∗∗	7.5 ± 0.3∗	133 ± 12
TNF*α* (18 hr)	361 ± 16∗∗	6.6 ± 0.6∗∗∗	167 ± 13∗∗
TNF*α* (36 hr)	286 ± 22∗	7.3 ± 0.2∗∗	143 ± 13∗
APO + TNF*α* (6 hr)	261 ± 15^#^	7.8 ± 0.4	116 ± 10
APO + TNF*α* (18 hr)	226 ± 15^##^	7.4 ± 0.4^#^	126 ± 17^#^
APO + TNF*α* (36 hr)	211 ± 14^#^	7.7 ± 0.2^#^	124 ± 11

Data are expressed as means ± S.E.M from *n* = 5 mice in each group. *P < 0.05, **P < 0.01, and ***P < 0.001 versus control; ^#^ *P* < 0.05 and ^##^ *P* < 0.01 versus TNF*α*.

## Abbreviations

TNF*α*: Tumor necrosis factor-*α*
NOX1: NADPH oxidase 1
NOXO1: NOX organizer 1
NOXA1: NOX activator 1
IBD: Inflammatory bowel disease
ROS: Reactive oxygen species
PMN: Polymorphonuclear neutrophils.
